# Supplementary material for: Venomix: a simple bioinformatic pipeline for identifying and characterizing toxin gene candidates from transcriptomic data
Source: PeerJ. 2018 Jul 31;6:e5361. doi: 10.7717/peerj.5361 (PMC6074769; doi:10.7717/peerj.5361)
Supplement: Supplemental Information 6 [file peerj-06-5361-s006.gz › FinalOutput_1E-20/Kunitz-type_serine_protease_inhibitor_Kunitz-1_1/finaltree.pdf]

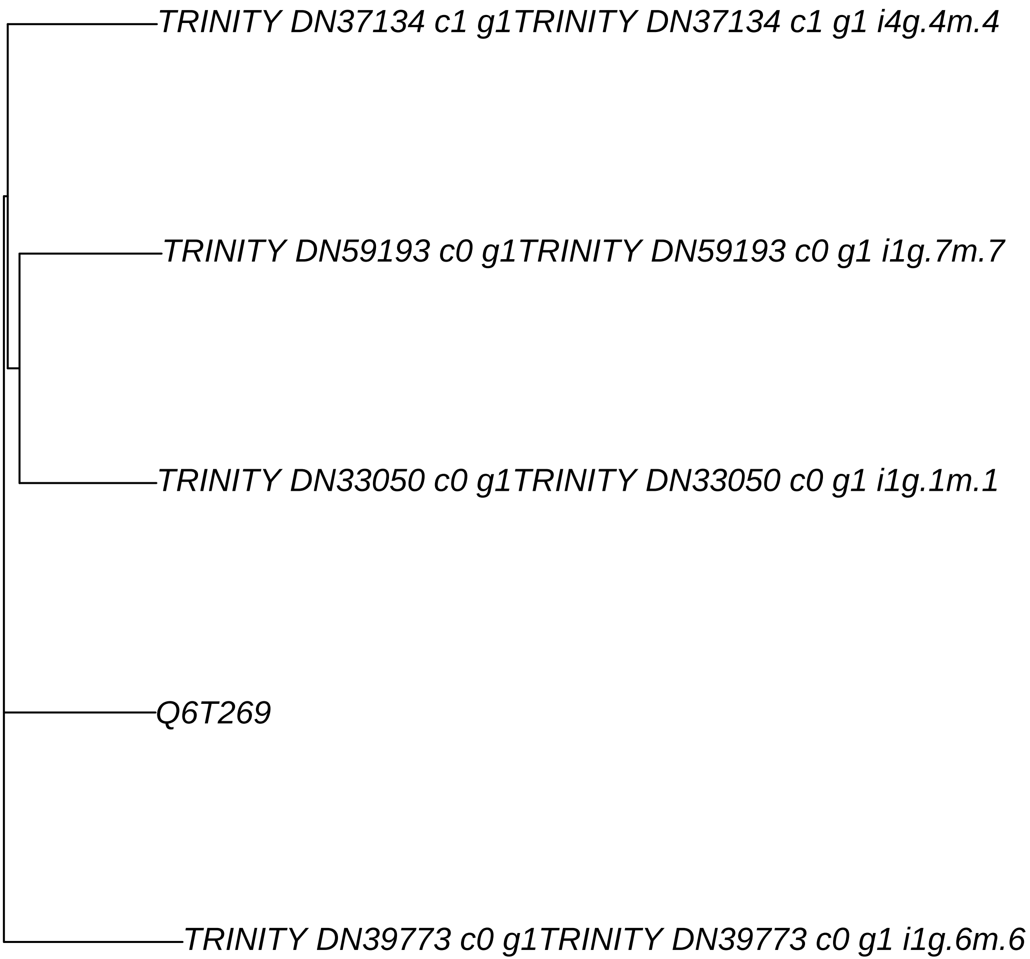

TRINITY DN37134 c1 g1TRINITY DN37134 c1 g1 i4g.4m.4
TRINITY DN59193 c0 g1TRINITY DN59193 c0 g1 i1g.7m.7
TRINITY DN33050 c0 g1TRINITY DN33050 c0 g1 i1g.1m.1
Q6T269
TRINITY DN39773 c0 g1TRINITY DN39773 c0 g1 i1g.6m.6
